# Primary Care Practitioner Perceptions on the Follow-up of Abnormal Cancer Screening Test Results

**DOI:** 10.1001/jamanetworkopen.2022.34194

**Published:** 2022-09-29

**Authors:** Steven J. Atlas, Anna N. A. Tosteson, Timothy E. Burdick, Adam Wright, Erica S. Breslau, Tin H. Dang, Amy J. Wint, Rebecca E. Smith, Kimberly A. Harris, Li Zhou, Jennifer S. Haas

**Affiliations:** 1 Division of General Internal Medicine, Massachusetts General Hospital, Harvard Medical School, Boston, Massachusetts; 2 The Dartmouth Institute for Health Policy and Clinical Practice, Geisel School of Medicine, Lebanon, New Hampshire; 3 Dartmouth Cancer Center, Dartmouth Health and Geisel School of Medicine at Dartmouth, Lebanon, New Hampshire; 4 Department of Community and Family Medicine, Dartmouth Health, Lebanon, New Hampshire; 5 Department of Biomedical Informatics, Vanderbilt University Medical Center, Nashville, Tennessee; 6 Division of Cancer Prevention and Control, National Cancer Institute, Rockville, Maryland; 7 Division of General Internal Medicine and Primary Care, Brigham and Women’s Hospital, Boston, Massachusetts

## Abstract

**Importance:**

Health care systems focus on delivering routine cancer screening to eligible individuals, yet little is known about the perceptions of primary care practitioners (PCPs) about barriers to timely follow-up of abnormal results.

**Objective:**

To describe PCP perceptions about factors associated with the follow-up of abnormal breast, cervical, colorectal, and lung cancer screening test results.

**Design, Setting, and Participants:**

Survey study of PCPs from 3 primary care practice networks in New England between February and October 2020, prior to participating in a randomized clinical trial to improve follow-up of abnormal cancer screening test results. Participants were physicians and advanced practice clinicians from participating practices.

**Main Outcomes and Measures:**

Self-reported process, attitudes, knowledge, and satisfaction about the follow-up of abnormal cancer screening test results.

**Results:**

Overall, 275 (56.7%) PCPs completed the survey (range by site, 34.9%-71.9%) with more female PCPs (61.8% [170 of 275]) and general internists (73.1% [201 of 275]); overall, 28,7% (79 of 275) were aged 40 to 49 years. Most PCPs felt responsible for managing abnormal cancer screening test results with the specific cancer type being the best factor (range, 63.6% [175 of 275] for breast to 81.1% [223 of 275] for lung; *P* < .001). The PCPs reported limited support for following up on overdue abnormal cancer screening test results. Standard processes such as automated reports, reminder letters, or outreach workers were infrequently reported. Major barriers to follow-up of abnormal cancer screening test results across all cancer types included limited electronic health record tools (range, 28.5% [75 of 263]-36.5%[96 of 263]), whereas 50% of PCPs felt that there were major social barriers to receiving care for abnormal cancer screening test results for colorectal cancer. Fewer than half reported being very satisfied with the process of managing abnormal cancer screening test results, with satisfaction being greatest for breast cancer (46.9% [127 of 271]) and lowest for cervical (21.8% [59 of 271]) and lung cancer (22.4% [60 of 268]).

**Conclusions and Relevance:**

In this survey study of PCPs, important deficiencies in systems for managing abnormal cancer screening test results were reported. These findings suggest a need for comprehensive organ-agnostic systems to promote timely follow-up of abnormal cancer screening results using a primary care–focused approach across the range of cancer screening tests.

## Key Points

**Question:**

What are the perceptions of primary care practitioners about the process, attitudes, knowledge, and satisfaction of following up on abnormal cancer screening test results?

**Findings:**

In this survey study of 275 primary care practitioners in 3 health systems, important deficiencies in systems for managing abnormal cancer screening test results that varied by specific cancer type were reported. Fewer than half of respondents reported being very satisfied with the process of managing abnormal cancer screening test results.

**Meaning:**

Results of this survey study suggest that health care systems need to develop a comprehensive primary care–focused approach across the range of cancer screening tests to ensure efficient and timely follow-up of abnormal results.

## Teaser

This survey study describes primary care practitioner perceptions about factors associated with the follow-up of abnormal breast, cervical, colorectal, and lung cancer screening test results.

## Introduction

Preventive screening for breast, cervical, colorectal, and lung cancer reduces cancer-specific mortality and is recommended by the US Preventive Services Task Force and other national guidelines.^1,2,3,4,5^ Achieving reductions in cancer-specific mortality starts with identifying and screening eligible individuals, and screening has been a focus of research and quality improvement efforts.^6^ Less attention has been paid to developing systematic processes to ensure the timely and appropriate follow-up of abnormal cancer screening test results (hereafter, *abnormal screens*).^7^ Studies suggest that high rates of timely follow-up, required to realize the maximal benefits of screening, are not being achieved.^7,8,9,10,11^

Barriers to follow-up of abnormal screens exist at multiple levels, including the patient, primary care practitioner (PCP), care team, specialist, and health system. For PCPs, there is uncertainty about responsibility, they lack systems to identify and track when patients are overdue for follow-up of an abnormal result,^12^ and they have insufficient time to perform outreach. Moreover, the transition from screening to diagnostic evaluation often involves coordination between PCPs and specialists and differs depending on the cancer screening test.^13,14^ Even though the PCP may not be the ordering clinician, PCPs still maintain the responsibility of managing the diagnostic evaluation of these multiple cancer screens with increasingly complex recommendations and time frames for test completion.^15^ The number of abnormal screens that PCPs are responsible for is large and may create medicolegal risks.^16,17,18^ However, few PCPs or their practices have integrated management systems to track abnormal screens and manage follow-up.

To develop a multilevel intervention to improve the follow-up of abnormal screens, we surveyed PCPs from participating practices prior to trial implementation. We sought to identify responsibility for managing abnormal results, systems and resources currently in place to help, the ease of scheduling follow-up care, barriers to ensuring timely follow-up, and knowledge of follow-up recommendations. Our goal was to identify deficiencies that could be addressed as part of a multilevel intervention focused on developing a system-level health informatics platform to identify and track abnormal screens, and enhancing clinical support team outreach.

## Methods

### Overview

This study was conducted as part of a National Cancer Institute–funded trial, mFOCUS (multilevel Follow-up of Cancer Screening).^19^ The trial was designed from the perspective of PCPs who take a whole-person approach with responsibility for all cancer screening tests endorsed by the US Preventive Services Task Force.^1,2,4,5^ Although the PCP may not order or perform a screening test and there are systems and policies designed to focus on a single type of cancer (eg, the Mammography Quality Standards Act for breast cancer screening results), PCPs are responsible for comprehensive cancer screening in collaboration with specialists or other clinicians involved in the diagnostic evaluation of abnormal screens. For these reasons, we sought to survey PCPs from sites participating in mFOCUS prior to initiating the trial. The study is registered at ClincialTrials.gov (NCT03979495) and has been reviewed and approved by the Mass General Brigham Institutional Review Board, which served as the central institutional review board. Return of the survey was deemed to be consent.

### Setting and Participants

Three primary care networks are participating in mFOCUS: 2 affiliated with Mass General Brigham in Massachusetts, Brigham and Women’s Hospital and Massachusetts General Hospital, and a third affiliated with Dartmouth Health in New Hampshire. Primary care physicians and advanced practice clinicians from 44 participating practice sites were identified from each of the networks. Physicians who were in residency training were excluded.

### Recruitment Protocol

The survey was sent to 485 PCPs prior to each network initiating enrollment for the trial. At the Brigham and Women’s Hospital and Massachusetts General Hospital sites, surveys were sent to 376 PCPs between February and March 2020. At Dartmouth Health sites, surveys were sent to 109 PCPs between September and October 2020. Primary care practitioners were initially sent an email invitation from the network’s medical director encouraging participant input about how they and their practice currently support the follow-up of abnormal screens. This recruitment invitation was followed by an email invitation from study investigators. Embedded within the email invitation was a hyperlink to the survey. PCP surveys were designed to be completed in 10 to 15 minutes and were self-administered using REDCap (up to 5 email contacts with a REDCap link over 4 weeks).^20^ As an incentive to complete the survey, PCPs were given a $50 gift card.

### Survey Content and Measures

The questionnaire content was adapted from the National Cancer Institute National Survey of Primary Care Physicians’ Cancer Screening Recommendations and Practices.^21^ Topics included perceptions of who is responsible for the follow-up of an abnormal result, mechanisms to track whether follow-up has been obtained, difficulty scheduling follow-up, clinician and patient barriers to follow-up, practice resources to assist with follow-up, and satisfaction with the process of managing an abnormal result. The PCP knowledge was assessed by providing scenarios for breast, cervical, colorectal, and lung cancers asking about how soon after a range of abnormal results (from less to more serious) would they typically recommend a follow-up test or referral for specialist consultation. Correct responses were based on guideline recommendations and local expert input. Clinician characteristics and experience items were investigator developed, including age, sex, specialty, number of office visits during a typical week, and prior litigation for failing to diagnose cancer were ascertained.

### Statistical Analysis

Reporting of survey response rates followed American Association for Public Opinion Research (AAPOR) reporting guideline for survey studies. Characteristics of PCPs and practices were summarized with descriptive statistics. We examined questionnaire responses stratified by respondent age, sex, specialty, and number of office visits during a typical week. Independent factors in questionnaire responses were assessed using logistic regression models. For questions that were separately asked for breast, cervical, colorectal, and lung cancer, independent factors were examined using logistic regression models with generalized estimating equations to account for clustering of multiple responses from the same respondent for different cancer types. Two-sided *P* values of .05 or less were considered statistically significant. All analyses used SAS version 9.4 (SAS Institute Inc).

## Results

### Study Population

Of 485 eligible PCPs, 275 (56.7%) completed the survey with 264 (54.4%) answering at least 90% of response items. Response rate differed by site (range, 34.9% [38 of 109] for Dartmouth Health to 71.9% [141 of 196] for Massachusetts General Hospital; *P* < .001). Participant characteristics are summarized in Table 1. Participant age and sex were similar across sites (overall, 28,7% [79 of 275] were aged 40-49 years and 61.8% [170 of 275] were female), but Brigham and Women’s Hospital and Massachusetts General Hospital PCPs were more likely to be general internists (74.0% [71 of 96] for Brigham and Women’s Hospital and 85.1% [120 of 141] for Massachusetts General Hospital vs 26.3% [10 of 38] for Dartmouth Health; *P* < .001) and see fewer patients per week (35.4% [34 of 96] for Brigham and Women’s Hospital and 25.5% [36 of 141] for Massachusetts General Hospital vs 55.3% [21 of 38] for Dartmouth Health seeing >51 patients per week; *P* = .007).

**Table 1.** Demographic Characteristics of 275 Survey Participants

PCP characteristic	No. (%)
Age range, y	
<40	62 (22.5)
40-49	79 (28.7)
50-59	75 (27.3)
≥60	56 (20.4)
Sex	
Female	170 (61.8)
Male	105 (38.2)
Clinician specialty	
General internal medicine (MD, DO)	201 (73.1)
Family medicine/Other (MD, DO)	35 (12.8)
Advance practice clinician (NP, PA)	35 (12.7)
Typical week, number of office visits	
<25	59 (21.5)
25-50	125 (45.5)
>51	91 (33.1)
Litigation for failing to diagnose cancer, yes	20 (7.3)

Abbreviations: DO, doctor of osteopathy; MD, medical doctor; NP, nurse practitioner; PA, physician assistant; PCP, primary care practitioner.

### Responsibility for Notifying and Managing Abnormal Screening Results

Primary care practitioners reported differences by cancer test in who they felt was responsible for notifying a patient of an abnormal screen (Table 2). For radiologic screening tests, the clinician interpreting mammograms was most commonly viewed as responsible for notifying the patient of an abnormal screen (70.9% [195 of 275]), but not for abnormal low dose computed tomography screens (7.6% [21 of 275]). For Papanicolaou tests and colonoscopies, most PCPs viewed that the clinician performing the screening test was responsible for notifying the patient (65.1% [179 of 275] for Papanicolaou tests and 84.4% [232 of 275] for colonoscopies). Despite differences in who was responsible for abnormal result notification, most PCPs still felt that they were responsible for managing abnormal screens, regardless of the cancer test (63.6% [175 of 275] to 81.1% [223 of 275]) (Table 2). Primary care practitioners reported that other members of the primary care team (eg, nurses, medical assistants, and administrative staff) were uncommonly involved in notification or managing abnormal results.

**Table 2.** Perceptions of Who is Responsible for Follow-up of Abnormal Screening Result by Cancer Type Among 275 Respondents

Responsibility for follow-up	Responsible for notifying patient, No. (%)^a^					Responsible for managing result follow-up, No. (%)^b^			
	Breast cancer, mammogram	Cervical cancer, Papanicolaou tests	Colorectal cancer		Lung cancer, low-dose computed tomography	Breast cancer, mammogram	Cervical cancer, Papanicolaou tests	Colorectal cancer	Lung cancer, low-dose computed tomography
			Colonoscopy	Stool cards					
PCP	88 (32.0)	148 (53.8)	80 (29.1)	171 (62.2)	210 (76.4)	175 (63.6)	183 (66.5)	192 (69.8)	223 (81.1)
Clinician									
Performing test	92 (33.5)	179 (65.1)	232 (84.4)	103 (37.5)	93 (33.8)	101 (36.7)	179 (65.1)	173 (62.9)	96 (34.9)
Interpreting test	195 (70.9)	3 (1.1)	31 (11.3)	1 (0.4)	21 (7.6)	132 (48.0)	4 (1.5)	19 (6.9)	23 (8.4)

Abbreviation: PCP, primary care practitioner.

^a^ “In your practice, who is usually responsible for notifying a patient about an abnormal result?”

^b^ “In your practice, whose responsibility is it to manage the follow-up evaluation of an abnormal result to ensure that a patient receives timely follow-up?”

In logistic regression models, the specific cancer type was associated with the highest adjusted odds ratios (aORs) of who was responsible for notifying patients and managing result follow-up (eg, the PCP was viewed as particularly responsible for notifying patients about lung vs breast cancer screening results, aOR 7.67 [95% CI, 5.48-10.7]; *P* < .001) (eTable 1 in the Supplement). Other factors associated with whether the PCP performing the test was responsible for managing result follow-up included the site, where Dartmouth Health respondents were less likely to say it was the PCP (aOR, 0.33 [95% CI, 0.17-0.65]), and specialty of the clinician (advanced practice clinicians vs internists, aOR, 0.44 [95% CI, 0.21-0.92]). Advanced practice clinicians were more likely to say it was the clinician performing the test (aOR, 2.88 [95% CI, 2.88-5.62]).

### Mechanisms to Follow-up Overdue Abnormal Screens

Primary care practitioners reported limited support for following up overdue abnormal screens. Few PCPs reported that their practice had an automated reporting mechanism to provide them or a member of their team with an alert when a patient is overdue for follow-up of an abnormal result independent of whether or not the patient was coming in for a visit (for cervical cancer, 14.5% [40 of 275] for PCP and 26.2% [72 of 275] for other team member) (eTable 2 in the Supplement). Standard processes to remind patients were reported by 21.8% (60 of 275) PCPs (sending reminder letters to patients for abnormal overdue lung cancer screening results) and by 23.6% (65 of 275) of PCPs (using population health manager or navigator to contact patients for abnormal overdue lung cancer screening results). In logistic regression models, respondents were less likely to report mechanisms to alert other team members and have standard reminder letters or population outreach for abnormal lung cancer results (16.7% [46 of 275]; aOR 0.47 [95% CI, 0.37-0.61] for reminder letter and aOR 0.61 [95% CI, 0.47-0.76] for population outreach for lung cancer; *P* < .001) (eTable 1 in the Supplement). Massachusetts General Hospital respondents were more likely to report having population health managers or patient navigators to support follow-up of abnormal results compared with those at Brigham and Women’s Hospital or Dartmouth Health (42.0% [59 of 141]; *P* < .001).

### Ease of Scheduling Follow-up Tests or Referrals

Fewer than half of PCPs reported that it was “very easy” to schedule a follow-up colposcopy for a positive cervical cancer screen (32.1% [87 of 271]) or specialty appointments for patients with an abnormal screen (15.1% [41 of 271] for lung cancer and 50.2% [137 of 273] for breast cancer) (Table 3). In logistic regression models, male respondents were less likely to report that it was very easy to schedule a breast biopsy (aOR, 0.41 [95% CI, 0.24-0.70) or appointment with a breast specialist for abnormal breast results (aOR, 0.39 [95% CI, 0.22-0.69]) (eTable 1 in the Supplement). Massachusetts General Hospital respondents were more likely to report that it was very easy to schedule any follow-up for abnormal breast results or a colonoscopy compared with those at Brigham and Women’s Hospital or Dartmouth Health (65.2% [92 of 141] for breast [*P* < .001] and 53.9% [76 of 141] for colonoscopy [*P* = .01]).

**Table 3.** Difficulty Scheduling Follow-up of Abnormal Screening Result

Ease of scheduling follow-up for patient with abnormal result^a^	No. (%)		
	Very easy	Somewhat easy	Somewhat or very difficult
Breast cancer			
Repeat mammogram or ultrasound	228 (83.5)	38 (13.9)	5 (1.8)
Breast biopsy	162 (59.6)	81 (29.8)	14 (5.1)
Appointment with breast surgeon	137 (50.2)	112 (41.0)	18 (6.6)
Cervical cancer			
Colposcopy	87 (32.1)	141 (52.0)	36 (13.3)
Appointment with gynecologist	74 (27.2)	128 (47.1)	68 (25.0)
Colorectal cancer			
Colonoscopy	124 (45.4)	107 (39.2)	42 (15.4)
Lung cancer			
Chest/PET CT	148 (54.4)	91 (33.5)	21 (7.7)
Appointment with lung specialist	41 (15.1)	113 (41.5)	98 (36.0)

Abbreviation: PET CT, positron emission tomography computed tomography.

^a^ “Overall, how easy or difficult is it to schedule follow-up tests for patients with an abnormal result?”

### Barriers to Follow-up of Overdue Abnormal Screens

Approximately a third of respondents reported that limited electronic health record tools were a major barrier for follow-up of abnormal screening results (range, 28.5% [75 of 263] for breast cancer to 36.5% [93 of 261] for colorectal cancer]), and approximately 20% reported limited staff to assist with follow-up was a major barrier for all cancer screening tests (17.6% [46 of 262] for breast cancer and 21.5% [56 of 260] for lung cancer) (Figure). Social barriers to receiving care, such as lack of transportation or time off from work, were felt to be a major barrier by 50.2% (137 of 273) of PCPs for patients with abnormal colorectal cancer screening results (*P* < .001 in logistic models) (eTable 1 in the Supplement), but not for other abnormal screens (16.5% [45 of 272] and 19.6% [53 of 270]).

**Figure.**
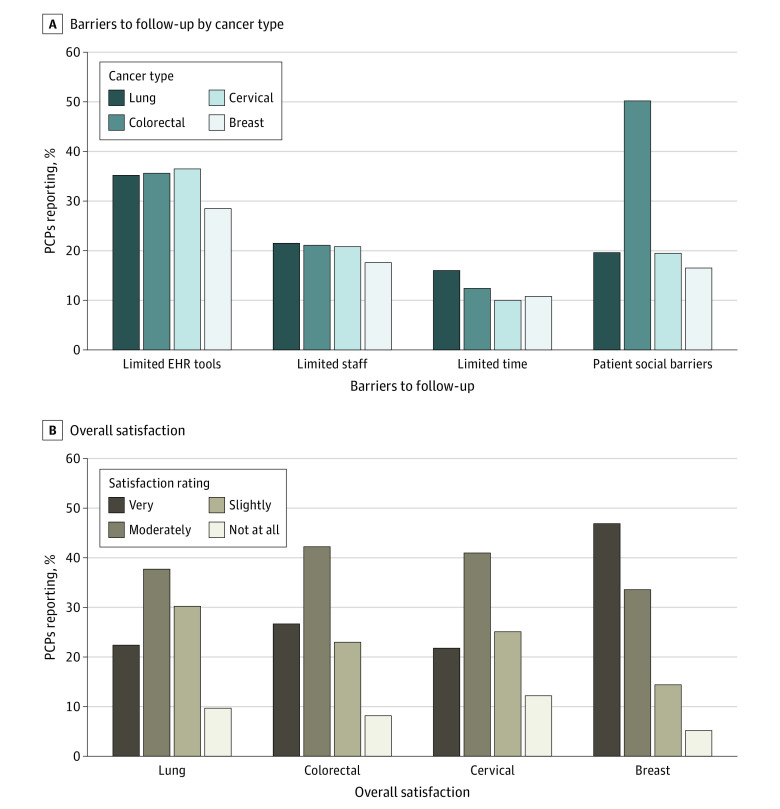
Barriers to Follow-up and Satisfaction With the Process Percent of primary care practitioners (PCPs) reporting “major barriers” in response to the question, “In your practice, are the following factors barriers to ensuring the follow-up of an abnormal result for your patients?” by cancer type (A). Percent of PCPs reporting “very satisfied” in response to the question, “Over the past year, how would you rate your overall satisfaction with the process for managing patients with an abnormal result?” by cancer type (B).

Other less commonly reported barriers are presented in eTable 2 in the Supplement. Limited availability of specialists and complex or difficult-to-follow guidelines or recommendations for diagnostic evaluations were more likely to be a major barrier for cervical (7.1% [19 of 269] for limited availability and 10.4% [28 of 270] difficult-to-follow guidelines) and lung cancer screening (7.5% [20 of 266] for limited availability and 10.9% [29 of 265] for difficult-to-follow guidelines), whereas insurance or financial barriers were more common for colorectal and lung cancer screening (15.3% [41 of 268] for colorectal and 16.3% [43 of 264] for lung cancer) and respondents at Dartmouth Health (32.4% [12 of 37]; *P* = .04) (eTable 1 in the Supplement).

### Clinician Knowledge of Guideline Recommendations

Knowledge of recommended follow-up intervals for abnormal cancer screening test results varied according to the cancer and the severity of the abnormal result (Table 4). Correct responses were lower for cervical (58.2% [160 of 275] for atypical squamous cells of undetermined significance with a negative human papilloma virus test result and 79.6% [219 of 275] for high-grade squamous intraepithelial lesion Papanicolaou tests, positive human papilloma virus test result and no history of abnormal screening results) and lung cancer (52.4% [144 of 275] for Lung Imaging Reporting and Data System category 3 and 68.7% [189 of 275] for Lung Imaging Reporting and Data System category 4b) and for abnormal screens with longer recommended follow-up intervals (38.2% [105 of 275] for single 1.5-cm adenomatous polyp). In logistic regression models, female respondents were more often correct for high-risk breast (92.4% [157 of 170] vs 78.6% [77 of 98] of male respondents) and cervical knowledge questions (90.0% [153 of 170] vs 62.2% [61 of 98] of male respondents; *P* < .005) (eTable 1 in the Supplement), and Massachusetts General Hospital respondents were more often correct for lung cancer knowledge questions (77.3% [109 of 141] vs 58.3% [56 of 96] and 63.2% [24 of 38]; *P* = .02).

**Table 4.** Knowledge of Recommended Follow-up of Abnormal Cancer Screening Test Results for Specific Clinical Scenarios From Survey Instrument

Clinical scenario: recommended window for follow-up for an abnormal result^a^	Correct response, No. (%)
Breast cancer screening	
55-y-old Woman with a mammogram with a BI-RADS 5 result: 3 mo	239 (86.9)
55-y-old Woman with a mammogram with a BI-RADS 3 result: 6 mo	143 (52.0)
Colorectal cancer screening	
60-y-old Man with positive FIT/FOBT: 3 mo	251 (91.3)
65-y-old Man with 10 or more adenomatous polyps: 1 y	141 (51.3)
70-y-old Man with a single 1.5-cm adenomatous polyp: 3 y	105 (38.2)
Cervical cancer screening	
33-y-old Woman with HSIL Papanicolaou test, HPV positive and no history of abnormal screening results: 3 mo	219 (79.6)
23-y-old Woman whose first Papanicolaou test shows ASCUS with a negative HPV test: 1 y	160 (58.2)
Lung cancer screening	
60-y-old Man with a low-dose lung CT with a Lung-RADS 4b result: 3 mo	189 (68.7)
60-y-old Man with a low-dose lung CT with a Lung-RADS 3 result: 6 mo	144 (52.4)

Abbreviations: ASCUS, atypical squamous cells of undetermined significance; BI-RADS, Breast Imaging Reporting and Data System; CT, computed tomography; FIT/FOBT, fecal immunochemical test/fecal occult blood test; HPV, human papilloma virus; HSIL, high grade squamous intraepithelial lesion; Lung-RADS, Lung Imaging Reporting and Data System.

^a^ “How soon after each of these abnormal results would you typically recommend a follow-up test or referral for specialist consultation?”

### Satisfaction With Processes to Manage Overdue Abnormal Screens

Primary care practitioners were asked to rate their overall satisfaction with the process of evaluating patients with an abnormal cancer screening result during the prior year. Fewer than half reported that they were very satisfied for each of the cancer screening tests (21.8% [59 of 271] for cervical cancer and 46.9% [127 of 271] for breast cancer) (Figure). Satisfaction was greatest for breast cancer (46.9% [127 of 271]) and lowest for cervical (21.8% [59 of 271]) and lung cancer (22.4% [60 of 268]). In logistic regression models, cancer type was statistically associated with the being very satisfied (eg, aOR 0.30 [95% CI 0.29-0.51] comparing screening for cervical cancer with screening for breast cancer; *P* < .001) (eTable 1 in the Supplement). Respondents from the Brigham and Women’s Hospital were less likely to be satisfied with the process of managing abnormal results (21.4% [18 of 84] vs 32.0% [8 of 25] for Dartmouth Health and 34.1% [47 of 138] for Massachusetts General Hospital; *P* = .008).

## Discussion

We surveyed PCPs affiliated with 3 practice networks in New England prior to enrolling individuals with overdue abnormal cancer screening test results in an intervention trial. The PCPs reported deficiencies in a range of processes to follow-up overdue abnormal results, suggesting the need for new models of care. Reported support varied among cancer types with breast cancer processes being rated highest and cervical and lung being lowest. Although PCPs variably reported that others were involved in the process of notifying patients with abnormal results, PCPs viewed themselves as being responsible for overall management regardless of the cancer test. Despite feeling responsible for managing abnormal results, there were knowledge gaps in recommended follow-up intervals, especially for findings with longer follow-up windows that are more likely to require PCP intervention. Few PCPs felt adequately supported by staff and electronic systems, and less than half of PCPs were very satisfied with the process of managing abnormal screening results for any cancer type.

Most prior surveys of practitioner attitudes and beliefs have focused on screening rather than abnormal result follow-up and on individual cancer tests rather than comprehensive cancer screening test follow-up.^18,22^ This focus on screening and individual cancer tests is seen despite evidence that inadequate follow-up of abnormal test results is well described and not limited to only certain screening tests.^7,23^ Given this situation, our findings are notable in showing that PCPs report deficiencies as being uniformly present but also variable in important ways among cancer screens. For example, follow-up of abnormal breast cancer screening results was viewed as being better than for other abnormal cancer screens and may reflect legislative efforts to ensure timely follow-up.^24^

Participants highlighted the complexities of tracking different abnormal cancer screening test results. Although participants reported that the person responsible for notifying patients about an abnormal screen depended on the specific test, most nonetheless viewed the PCP as being responsible for managing the abnormal screen. Prior efforts focused on improving follow-up for a single cancer test take a more specialist-oriented approach and may result in optimal systems that vary among the different screening tests.^7,25^ Our results may be viewed as supporting an approach whose goal is to develop tracking systems and protocols that are similar regardless of the specific cancer screening test rather than customizing to the specific test and result.

The multiple deficiencies reported by PCPs, including varying roles among clinicians, inadequate information technology and personnel support systems, and knowledge gaps, point to the need for multilevel interventions to support patients and PCPs, as well as integration efforts involving specialists. If PCPs are going to bear responsibility for at least overseeing the follow-up of abnormal screens, then technology solutions should function similarly for the different screening test results. Such efforts should also support other team members who may be involved in only limited aspects of ensuring timely follow-up. Initial systems may begin by providing fail-safe oversight if existing cancer-specific protocols are in place. This oversight is especially useful when there is variable support across cancer types. If such comprehensive systems prove effective, one could then transition them to being involved sooner in the follow-up process.

### Limitations

This study has limitations. These results may not be generalizable to PCPs in other practice networks. Given that the resources available in the studied networks may exceed those in other practices, it is possible that these results may overstate the support available to PCPs more broadly. Thus, our findings are more likely to understate the nature of the deficiencies identified. Our response rate is similar to other surveys of health care professionals, and lower rates decrease the validity of the reported findings.^26^ Our findings are based on PCP perceptions and PCPs may not know all resources available to them. It is also possible that responses may have overestimated available reminder processes because PCPs may consider support for routine screening efforts to be applicable to managing an abnormal screen, even if this is not the case. For example, electronic health record reminders to obtain overdue routine cancer screening tests may not be automatically updated when an abnormal result occurs that warrants diagnostic testing or shorter subsequent follow-up intervals. In addition, these PCP responses are likely to be affected by the COVID-19 pandemic. Survey of PCPs at Brigham and Women’s Hospital and Massachusetts General Hospital occurred just prior to the first wave of the pandemic. Surveys at Dartmouth Health occurred in September and October of 2020. It is likely that the deficiencies have only been magnified by the pandemic and that improved systems are needed more than ever to decrease barriers to follow-up of abnormal screens.

## Conclusions

In this survey study of PCPs from 3 primary care networks, they reported important deficiencies in managing the follow-up of all abnormal cancer screening test results and identified particular areas where process varied among the cancer types. Despite differences among the cancer screening tests, most PCPs viewed themselves as being responsible for managing all abnormal screens. These findings suggest the need for comprehensive systems to promote timely follow-up of abnormal cancer screening results using a primary care–focused approach across the range of preventive cancer tests.

## Notes

Supplement. **eTable 1.** Independent Predictors (Cancer Type, PCP and Practice Characteristics) of Questionnaire Responses Using Multivariable Logistic Regression Models **eTable 2.** Mechanisms to Alert PCPs, Standard Processes to Remind Patients that They are Overdue, and Barriers to Follow-up of Abnormal Screening Results by Cancer Type
