# Supplementary material for: Primary Care Practitioner Perceptions on the Follow-up of Abnormal Cancer Screening Test Results
Source: JAMA Netw Open. 2022 Sep 29;5(9):e2234194. doi: 10.1001/jamanetworkopen.2022.34194 (PMC9523497; doi:10.1001/jamanetworkopen.2022.34194)
Supplement: Supplement. — eTable 1. Independent Predictors (Cancer Type, PCP and Practice Characteristics) of Questionnaire Responses Using Multivariable Logistic Regression Models eTable 2. Mechanisms to Alert PCPs, Standard Processes to Remind Patients that They are Overdue, and Barriers to Follow-up of Abnormal Screening Results by Cancer Type [file jamanetwopen-e2234194-s001.pdf]

Supplemental Online Content

Atlas SJ, Tosteson ANA, Burdick TE, et al. Primary care practitioner perceptions on the follow-up of abnormal cancer screening test results. *JAMA Netw Open.* 2022;5(9):e2234194. doi:10.1001/jamanetworkopen.2022.34194

eTable 1. Independent Predictors (Cancer Type, PCP and Practice Characteristics) of Questionnaire Responses Using Multivariable Logistic Regression Models

eTable 2. Mechanisms to Alert PCPs, Standard Processes to Remind Patients that They are Overdue, and Barriers to Follow-up of Abnormal Screening Results by Cancer Type

This supplemental material has been provided by the authors to give readers additional information about their work.

© 2022 Atlas SJ et al. *JAMA Network Open*.

eTable 1. Independent Predictors (Cancer Type, PCP and Practice Characteristics) of Questionnaire Responses Using Multivariable Logistic Regression Models [a]

Predictor (reference), aOR (95%CI)	Cancer Type (breast)			PCP Age (≤50)	PCP Sex (Female)	PCP Specialty (Internal Medicine)	# Patients (<50/week)	Site (MGH)
	Cervical	Colorectal	Lung					
**Responsible for notifying patient (%)**								
PCP	2.60 (1.95-3.48)	NS	7.67 (5.48-10.7)	-	1.72 (1.15-2.57)	0.44 (0.20-0.96) [b]	NS	0.40 (0.21-0.77) [d]
Provider performing test	3.80 (2.71-5.33)	11.43 (7.7-17.0)	NS	0.67 (0.46-0.96)	-	4.26 (2.27-8.0) [b]	-	-
Provider interpreting test	0.00 (0.00-0.01)	0.05 (0.04-0.08)	0.03 (0.02-0.05)	-	1.58 (1.00-2.51)	-	-	-
**Responsible for managing result follow-up (%)**								
PCP	NS	1.36 (1.07-1.73)	2.67 (2.00-3.56)	-	-	0.44 (0.21-0.92) [b]	-	0.33 (0.17-0.65) [d]
Provider performing test	3.32 (2.50-4.40)	3.01 (2.31-3.91)	NS	-	-	2.88 (1.48-5.62) [b]	-	-
Provider interpreting test	0.01 (0.01-0.04)	0.08 (0.05-0.12)	0.09 (0.06-0.15)	-	-	0.41 (0.17-1.00) [b]	NS	-
**Automated report alerts for review to**								
PCP	NS	1.13 (1.00-1.28)	NS	-	-	0.28 (0.09-0.84) [b]	-	-
Other team member	NS	NS	0.61 (0.48-0.77)	-	-	3.45 (1.62-7.35) [c]	-	0.31 (0.11-0.85) [d]
**Standard reminder processes**								
Reminder letter sent by mail or patient portal	0.80 (0.65-0.98)	0.69 (0.58-0.82)	0.47 (0.37-0.61)	-	-	NS	NS	NS
Automated phone call or text	-	-	-	-	-	NS	-	NS
Population health manager or patient navigator	NS	NS	0.60 (0.47-0.76)	-	-	-	-	0.45 (0.27-0.76) [e]; 0.14 (0.04-0.47) [d]

© 2022 Atlas SJ et al. *JAMA Network Open*.

Predictor (reference), aOR (95%CI)	Cancer Type (breast)			PCP Age (≤50)	PCP Sex (Female)	PCP Specialty (Internal Medicine)	# Patients (<50/ week)	Site (MGH)
	Cer-vical	Colo-rectal	Lung					
**Ease of scheduling follow-up for abnormal screens, very easy**								
Repeat mammogram or ultrasound	NA	NA	NA	-	NS	-	-	0.38 (0.18-0.78) [e]
Breast biopsy	NA	NA	NA	-	0.41 (0.24-0.70)	-	-	0.37 (0.21-0.65) [e]
Appointment with breast surgeon	NA	NA	NA	NS	0.39 (0.22-0.69)	-	-	0.16 (0.09-0.30) [e]
Colposcopy	NA	NA	NA	-	-	2.64 (1.15-6.07) [b]	NS	0.49 (0.26-0.93) [e]
Appointment with gynecologist	NA	NA	NA	-	-	3.07 (1.33-7.06) [b]	NS	NS
Colonoscopy	NA	NA	NA	-	NS	-	-	0.44 (0.25-0.76) [e]
Chest/ PET CT	NA	NA	NA	-	NS	-	-	-
Appointment with lung specialist	NA	NA	NA	-	2.06 (1.05-4.03)	-	-	-
**Barriers to Follow-up of Abnormal Screens**								
Limited EHR tools to facilitate management	1.47 (1.25-1.72)	1.39 (1.18-1.65)	1.38 (1.17-1.63)	-	-	NS	0.44 (0.25-0.76)	-
Limited staff to assist in my practice	1.23 (1.07-1.41)	1.20 (1.02-1.42)	1.30 (1.10-1.54)	0.48 (0.26-0.88)	-	-	-	NS
Limited time to discuss	NS	NS	1.54 (1.23-1.94)	-	-	-	-	-
Patients have social barriers to receiving care	1.20 (1.06-1.36)	5.02 (3.72-6.78)	1.24 (1.06-1.46)	-	-	-	-	-
Guidelines/recommendations difficult/complex	2.39 (1.23-4.65)	NS	2.52 (1.48-4.31)	-	-	-	-	2.68 (1.19-6.05) [d]
Limited availability of specialist	4.09 (1.80-9.30)	2.71 (1.23-5.96)	4.44 (2.05-9.60)	0.35 (0.16-0.75)	-	0.22 (0.05-0.99) [c]	-	-
Patients do not understand importance of timely follow-up	2.16 (1.50-3.11)	2.82 (1.99-4.00)	2.13 (1.50-3.02)	-	-	NS	-	NS
Patients have insurance/financial barriers	NS	1.53 (1.19-1.97)	1.64 (1.25-2.14)	-	NS	-	NS	3.53 (1.56-7.98) [d]

© 2022 Atlas SJ et al. *JAMA Network Open*.

Predictor (reference), aOR (95%CI)	Cancer Type (breast)			PCP Age (≤50)	PCP Sex (Female)	PCP Specialty (Internal Medicine)	# Patients (<50/ week)	Site (MGH)
	Cer-vical	Colo-rectal	Lung					
**Knowledge of Recommended Timing of Follow-up**								
55 year old woman with a mammogram with a BIRADS 5 result: 3 months	NA	NA	NA	-	0.29 (0.14-0.63)	-	-	0.36 (0.16-0.83) [e]
55 year old woman with a mammogram with a BIRADS 3 result: 6 months	NA	NA	NA	NS	NS	-	-	-
60 year old man with positive FIT/FOBT: 3 months	NA	NA	NA	NS	-	NS	-	0.22 (0.07-0.65) [e]
65 year old man with 10 or more adenomatous polyps: 1 year	NA	NA	NA	-	-	NS	-	-
70 year old man with a single 1.5cm adenomatous polyp: 3 years	NA	NA	NA	-	-	-	-	0.54 (0.31-0.94) [e]
33 year old woman with HSIL Pap, HPV+ and no history of abnormal screening results: 3 months	NA	NA	NA	-	0.19 (0.10-0.37)	-	-	NS
23 year old woman whose first Pap shows ASCUS with a negative HPV test: 1 year	NA	NA	NA	-	-	-	-	2.32 (1.02-5.26) [d]
60 year old man with a low dose lung CT with an	NA	NA	NA	NS	-	NS	-	0.49 (0.27-0.89) [e]

© 2022 Atlas SJ et al. *JAMA Network Open*.

LRADS 4b result: 3 months								
60 year old man with a low dose lung CT with an LRADS 3 result: 6 months	NA	NA	NA	NS	-	NS	-	0.39 (0.23-0.69) [e]
Satisfaction with the Process of Managing Abnormal Screens Results, very satisfied	0.30 (0.22-0.40)	0.38 (0.29-0.51)	0.32 (0.24-0.42)	NS	NS	-	1.77 (1.09-2.86)	0.44 (0.27-0.74) [e]

aOR – adjusted odds ratio; PCP – primary care practitioner; MGH – Massachusetts General Hospital; "-" variable did not meet criteria for model entry; NS – Variable entered into model but not statistically significant; NS – cancer type not included in model; PET CT – Positron emission tomography computerized tomography; EHR – electronic health record; BIRADS – Breast Imaging Reporting and Data System; FIT/FOBT – fecal immunochemical test/fecal occult blood test; HSIL – high grade squamous intraepithelial lesion; HPV – human papilloma virus; ASCUS – atypical squamous cells of undetermined significance; CT – computerized tomography; LRADS - Lung Imaging Reporting and Data System

[a] Logistic regression with GEE models including potential predictors significant at 0.15 level in the bivariate analysis presented as adjusted odds (aOR) ratio with 95% confidence intervals (95%CI)

[b] PCP specialty: nurse practitioner/physician assistant

[c] PCP specialty: family medicine provider

[d] Site: Dartmouth Health

[e] Site: Brigham and Women's Hospital

© 2022 Atlas SJ et al. *JAMA Network Open*.

eTable 2. Mechanisms to Alert Primary Care Practitioners, Standard Processes to Remind Patients that They are Overdue, and Barriers to Follow-up of Abnormal Screening Results by Cancer Type

Mechanisms to Alert PCPs, N (%)	Breast cancer	Cervical cancer	Colorectal cancer	Lung cancer
Automated report alerts for review to [a]				
PCP	36 (13.1)	40 (14.5)	40 (14.5)	34 (12.4)
Other team member	68 (24.7)	72 (26.2)	66 (24.0)	46 (16.7)
Standard reminder processes [b]				
Reminder letter sent by mail or patient portal	99 (36.0)	86 (31.3)	78 (28.4)	60 (21.8
Automated phone call or text	7 (2.5)	5 (1.8)	5 (1.8)	4 (1.5)
Population health manager or patient navigator	92 (33.5)	94 (34.2)	98 (35.6)	65 (23.6)
Barriers to follow-up, [c] responding “major barrier”				
Guidelines/recommendations difficult/complex	12 (4.5)	28 (10.4)	13 (4.9)	29 (10.9)
Limited availability of specialist	5 (1.9)	19 (7.1)	13 (4.9)	20 (7.5)
Patients do not understand importance of timely follow-up	25 (9.3)	47 (17.7)	60 (22.4)	46 (17.6)
Patients have insurance/financial barriers	29 (10.9)	24 (9.0)	41 (15.3)	43 (16.3)

PCP – primary care practitioner

[a] “Does your practice have a mechanism that alerts you or other care team members when a patient is overdue for follow-up of an abnormal result?”

© 2022 Atlas SJ et al. *JAMA Network Open*.

[b] “Does your practice have a standard process to remind patients that they are overdue for follow-up of an abnormal result?

[c] “In your practice, are the following factors barriers to ensuring the follow-up of an abnormal result for your patients?

© 2022 Atlas SJ et al. *JAMA Network Open*.
